# Properties of Texturized Vegetable Proteins from Edible Mushrooms by Using Single-Screw Extruder

**DOI:** 10.3390/foods12061269

**Published:** 2023-03-16

**Authors:** Sunantha Ketnawa, Saroat Rawdkuen

**Affiliations:** 1 Food Science and Technology Program, School of Agro-Industry, Mae Fah Luang University, Chiang Rai 57100, Thailand; 2 Unit of Innovative Food Packaging and Biomaterials, School of Agro-Industry, Mae Fah Luang University, Chiang Rai 57100, Thailand

**Keywords:** plant-based meat, texturized vegetable protein, King Oyster, Pheonix, single-screw extrusion

## Abstract

This research aimed to determine the feasibility of using mushrooms as an alternative ingredient in texturized vegetable protein (TVP) production using a single-screw extruder. TVPs from King Oyster (TVP-KO) and Pheonix mushroom (TVP-PH) were successfully developed and characterized. The visual appearance of TVP was reddish-brown, with a distinct roasted mushroom-soybean aroma. When rehydrated and cooked, both TVPs provided a minced meat-like appearance and chewy meat texture comparable to commercial TVP (TVP-Com); however, they had inferior water and oil holding and rehydration capacities. TVPs contained comparable protein content to TVP-Com (45–47 wt%), slightly lower carbohydrate content (33–36 wt% vs. 39 wt%), and ash (3–4 wt% vs. 8 wt%), but higher lipid content (7–8 wt% vs. 0.84 wt%) than TVP-Com. Sai-aua prepared from TVP-KO gained the highest overall acceptability. Mushrooms proved to be a potential source for TVP production due to their availability, low cost, nutritional value, and health benefits. Moreover, this finding helps add value to traditional meat products, which offer an opportunity for developing non-animal products with satisfactory sensory properties and low cost. In addition, the study would provide scientific resources for developing plant-based meat products that address health awareness and economic and environmental sustainability concerns.

## 1. Introduction

The trends of plant-protein-based meat analogs have been driven by the combined effects of a growing global population, awareness of the sustainability of animal protein, and societal and environmental concerns. Not only those mentioned but also consumer health considerations on plant protein-based meat analogs that contribute to the health benefits, such as being low in saturated fats and sodium, cholesterol-free, and an excellent source of comparable protein to animal meat [1,2,3]. Between 2018 and 2026, the global, Asia-Pacific, and European markets for plant-based meat are anticipated to expand at CAGRs of 14.8, 15.9, and 14.4%, respectively [4]. These challenges encourage the development of plant-based meat analogs that replicates the aesthetic qualities (structure, flavor, and appearance) of animal meat. These studies attempted to create plant-based meat analogs from various protein sources (e.g., soybean, green pea, peanut, rice bran, oilseed, cereal, and mycoprotein) [2]. Kyriakopoulou et al. [5] and Webb et al. [6] summarized the sources of the plant-based proteins most widely used in meat analogs in recent years are soy protein and wheat gluten, while other sources have also been used in meat analogs, including legumes from peas, faba beans, kidney beans, mycoprotein, and mushrooms [6].

Mushrooms have been used as meat substitutes in human diets due to their high macronutrient (protein and fiber) and micronutrient (essential amino acids, vitamins, and essential minerals) content, as well as their low fat, sodium, and calorie content [7,8]. Mushrooms have been substituted in developing texturized vegetable protein (TVP), beef/chicken patties, sausage, and nuggets to create healthier protein foods with an appealing appearance, flavor, and texture [8,9]. Recent studies have reported the use of mushrooms in meat products due to their distinctive flavors and health-promoting properties [10], but few have examined the use of mushrooms as the primary ingredient in TVP. According to Riaz [11], TVP or meat extender is a plant protein-based product produced from the extrusion processing of defatted soy flour or flakes and soy concentrates. TVP expands as it exits the extruder die and forms a random, spongy meat-like structure when hydrated, imitating the chewy texture of meat. The size and speed of the extruder’s cutting knife determine the size and shape of the extruded material. Typically, they are purchased in a dry form (6–10% moisture) in sizes ranging from small flakes or granules of approximately 2 mm or cubes of approximately 6–20 mm to large “steaks” that are 12 mm thick by 80 mm wide by 120 mm long [11,12].

Different processing techniques, e.g., single screw extrusion [1], twin-screw extrusion [7,8,9], Couette cell technology, shearing, and electrospinning [5], were applied to develop plant protein-based meat alternatives. The main challenges were the operation to achieve the right texture, appearance, and nutrient content of meat analogs. In a previous study, Mohamad et al. (2020) [1] reported the combined effect of low-grade oyster mushroom addition and the extrusion setting parameters on the properties of soy protein-based meat analogs were assessed via a single-screw extrusion. Another group of researchers investigated edible mushrooms and soybean protein isolate (SPI) to prepare a fibrous meat analog using thermos-extrusion and the extruded mushroom-based meat analog as a meat substitute, as well as different formulations for non-meat sausage [13,14]. In addition, Lee et al. [15] reported the production of rice bran protein and soy protein isolate (SPI) through low-moisture single-screw extrusion cooking.

As mentioned above, the different plant-based meat processing methods that have recently been developed are more expensive than authentic meat products. This is because they require more technology, technical know-how, specific machinery, raw material yield, expensive binders or additives, and a larger production scale. Currently, the food industry cares a lot about processing costs and the environment, and it is expected that new ways will be found to reduce processing costs and waste losses. Apart from that, one of the advantages of using mushrooms for this purpose is their compatibility with meat products because of their umami flavor and fibrous meat-like texture [1,8,10,16]. Moreover, the advantages of alternative sources such as mushrooms promote not only their healthy aspects but also their affordability, availability, scalability, and sustainability [7]. Thus, in this study, the feasibility of developing TVPs using mushrooms as a main ingredient via conventional single-screw cold extrusion. The developed conventional cold extrusions, which are (1) mixing and hydration, (2) mechanical treatment for texturization, and (3) drying while looking to improve the taste, texture, and storage shelf-life stability of plant-based meats with intelligible methods without costly additives and investment. Therefore, the set objectives were to (1) select two potential mushrooms to be the main ingredients for the development of TVPs, (2) determine the feasibility of using mushrooms as main ingredients for the development of TVPs via a conventional single-screw cold extrusion, and (3) evaluate the physicochemical properties (i.e., cooking loss, water-holding capacity, oil holding capacity, rehydration capacity, and bulk density), texture profile, and microstructure of TVPs, and (4) prepare as a meat substitute in Thai Northern-style sausage (Sai-aua) and analyze organoleptic properties.

## 2. Materials and Methods

### 2.1. Materials

King Oyster (*Pleurotus eryngii*, KO) from Rayong mushroom farm and VV Biotec. Co., Ltd., Rayong, Thailand was distributed by the supermarket, Makro, Chiang Rai, Thailand. Pheonix or Indian oyster (*Pleurotus pulmonarius*, PH) was purchased from mushroom farm, Tha-sud and Phan, Chiang Rai, Thailand. Soybean protein isolate (SPI) and vital wheat gluten (WG) were purchased from Krungthep Chemipan Co., Ltd., Bangkok, Thailand. Chickpea flour purchased was from purchased HuglamoolFarm, Ubon Ratchathani, Thailand. Additives such as yeast extract and beet juice powder were acquired from Value Industrial Products Co., Ltd., Bangkok, Thailand and Narah Co., Ltd., Chiang Mai, Thailand, respectively. Commercial TVP (TVP-Com) (long granule shape, size 5 mm length and 2 mm width) was purchased from FoodTech Products, Co., Ltd., Prathumthani, Thailand.

### 2.2. Reagents

All reagents used were of analytical grade. The distilled water produced from a water purification system was used in all analyses and experiments.

### 2.3. Processing of Dehydrated Mushroom Powder

The fresh King Oyster (KO) and Phoenix (PH) mushrooms were washed to remove dirt and foreign particles, and they were left in a strainer for 15 min to remove excess water. For KO, the mushroom body was cut horizontally at about 3 mm in thickness, whereas PH was manually shredded horizontally, then boiled in boiling water for 5 min. They were placed in a strainer for 15 min to cool before removing excess water again with a vegetable spinner. They were then chopped into coarse pieces using a meat grinder (SOKANY^®^, Model SK-312, Yiwu, China). The coarsely minced mushroom was seared by pan without oil at 70–85 °C and monitored by a handheld thermometer and needle probe kit Traceable^®^6460, Webster, TX, USA) for 45–60 min until the moisture content was around 65 wt%. The dehydrated minced mushroom was left to cool to room temperature and kept in a resealable low-density polyethylene zipper storage bag (Ziploc^®^, San Diego, CA, USA) and stored at −20 °C until being used as a starting material and further analyses.

### 2.4. Preparation of Texturized Vegetable Protein

A mixture of ingredients was prepared and weighed into the bowl according to Table 1. The ratios of mixtures were concealed and not allowed to be provided in the paper due to matters regarding Mae Fah Luang University’s intellectual property. The dehydrated mushroom from Section 2.3 was soaked in distilled water, which had previously been mixed with beet juice powder, for 15 min. The mushroom-infused beet juice from the previous step was poured into a mixer bowl and mixed using a whisk with a mixer (Model 5KPM5EWH, KITCHENAID^®^, St. Joseph, MI, USA) while gradually adding dried ingredient powder, including soy protein isolate, chickpeas, yeast extract, and vital wheat gluten while mixing for 10 min before adding canola oil. The mixture was mixed well for 15 min and subsequently rested at room temperature for another 10 min. After the designated time, the mixture was formed into tiny chunks or granules with a size of 3 mm × 3 mm using a tabletop screw meat grinder (SOKANY^®^, Model SK-312, Yiwu, China). Within this step, wet TVP analogs from two mushrooms were shaped by a conventional single-screw cold extrusion. The wet TVP analogs were dried using a tabletop electric oven (OTTO, CO-707, Supor Co., Ltd., Hangzhou, China) at 100 °C for 15 min, turning the TVP analogs every 2 min, and continued drying at 150 °C for 10 min, turning the mixture over every 2 min to allow the mixture to dry thoroughly. The moisture content of dried TVP analogs was determined and controlled to be around 8–10 wt%. The TVP analogs were left at room temperature, some of which were tested for moisture content, texture profile analysis, appearance, and color, while others were kept in a polyethylene zipper storage bag (Ziploc^®^, San Diego, CA, USA) and stored at −20 °C until being used as a starting material and undergoing further analyses. Commercial TVP was marked as TVP-Com, kept in the same condition, and used for comparison with TVP-KO and TVP-PH. This step was performed in triplicate to ensure the accuracy of the replication of trials.

### 2.5. Proximate Composition of Selected Mushrooms and TVPs

Different dried mushroom powder and TVPs were ground into powder using a blender (PHILIPS, ProBlend, HR2041/30/AC, Taizhou, China) and sifted through a 60 mesh sieve to obtain a homogeneous fine powder. The Association of Official Analytical Chemists (AOAC) [17] was used to quantify moisture, protein, lipid, and ash. The water activity of each TVP was measured using a water activity meter (Aqualab 4TE/PRE, METER Group Inc., Pullman, WA, USA). The moisture content of each TVP was determined using an oven-drying method at 105 °C. Ash content was determined by incinerating around 3 g of sample powder in a furnace (Fisher Scientific, Waltham, MA, USA) at 575 °C overnight. The protein content of all sample powders was determined for total nitrogen content based on the Kjeldahl method, with a conversion factor of 6.25. Soxhlet apparatus was utilized to measure the fat content using petroleum ether as an extracting solvent. All proximate compositions were analyzed in triplicate and reported as a percentage of dry weight (wt%, d.b.). The total carbohydrate content was calculated by deducting the total protein, fat, and ash contents from 100%, as shown in Equation (1):

Total carbohydrate (wt%, d.b.) = 100 − (protein + ash + fat) (wt%, d.b.)(1)

### 2.6. Amino Acid Compositions

For analysis of amino acid compositions, only KO and PH mushrooms were selected. Central Laboratory, Ltd., Muang, Chiang Mai, Thailand, analyzed the amino acid compositions of dried mushroom powders using the in-house method TE-CH-372 based on the Official Journal of the European Journal of communities, L257/16 by amino acid analyzer technique and reported the results as mg/100 g sample, d.b.

### 2.7. Physicochemical Determination

#### 2.7.1. Color Analysis, pH, and Water Activity

The color of each TVPs was determined using a portable colorimeter (CM-600d1, Konica Minolta Inc., Tokyo, Japan) (10° standard observers, illuminant D65) that was calibrated using a standard white plate according to the reference CIE 1976 L* a* b* color space [18]. The CIE L* a* b* values of the samples were recorded at ten random locations on the sample surface of TVPs. The parameters L* indicated the lightness of the sample from blackness (L* = 0) to whiteness (L* = 100). The a* indicated red (a* is positive) and green (a* is negative) of the sample. The b* indicated yellow (b* is positive) and blue (b* is negative) of the sample.

The pH of the samples was measured with a digital pH meter after homogenization of the sample with distilled water in the ratio of 1:1 (*w*/*v*). The water activity (a_w_) of all samples was analyzed in triplicate to evaluate their microbial stability. A water activity meter (AquaLab Ver 3TE, Decagon Devises, Pullman, WA, USA) was used.

#### 2.7.2. Visual Surface Appearance, Microstructure by Scanning Electron Microscopy (SEM)

Visual appearance: TVPs were photographed with a mobile phone (iPhone X, Apple^®^, Cupertino, CA, USA) under a portable photo studio box equipped with high-brightness LED modules.

Microstructure: Scanning electron microscopy (SEM) analysis was conducted to observe fibrous structures and air cell formations in TVPs. The microstructure of TVP was observed by a scanning electron microscope (TESCAN, MIRA, TESCAN ORSAY HOLDING, Brno, Czech Republic). Sample pieces were prepared and mounted onto aluminum stubs using double-sided carbon tape and then coated with gold particles. The images were photographed at magnification levels of 500×, 2000×, and 5000×, respectively, with an accelerating voltage of 10 keV features a maximum resolution of 200 µm.

#### 2.7.3. Water-Holding Capacity (WHC) and Oil Holding Capacity (OHC)

Minor modifications were made to the previous method described by Hong et al. [19] to conduct water/oil holding capacity (WHC/OHC) tests. For WHC, 0.5 g (W_0_) of ground dried TVP analog sample was dispersed in 5 mL deionized (DI) water in a 15 mL centrifuge tube that had been pre-weighed (W_1_). The mixture was thoroughly vortexed and allowed to rest for 5 min at room temperature. The supernatant was discarded after 10 min of centrifugation at 8000× *g*, and the tube containing the residue was inverted and allowed to rest for 5 min prior to reweighing (W_2_). For OHC, 0.5 g (O_0_) of each sample was thoroughly mixed with 5 mL canola oil in a 15 mL centrifuge tube that had been pre-weighed (O_1_). The mixture was allowed to stand at room temperature for 30 min before being centrifuged at 8000× *g* for 10 min. After removing the oil, the tube containing the protein sediment was inverted for 10 min to drain the excess oil, then reweighed (O_2_). Each sample was performed three times. The WHC and OHC were calculated by using the Equations (2) and (3), respectively, and expressed as grams of water and oil absorbed per gram of sample, respectively.

(2) $$\mathrm{WHC}\;(\frac{{g\;H}_{2}O}{g\;\mathrm{sample}})=\frac{\left(W_{2}{-W}_{1}{-W}_{0}\right)}{W_{0}}\;$$

(3) $$\mathrm{OHC}\;\left(\frac{g\;\mathrm{oil}}{g\;\mathrm{sample}}\right)=\frac{\left(O_{2}{-O}_{1}{-O}_{0}\right)}{O_{0}}$$

#### 2.7.4. Rehydration Capacity and Rehydration Yield

Minor modifications were made to the previous method described by Hong et al. [19] to conduct the analysis of rehydration capacity and yield. Twenty grams of TVPs were rehydrated in 300 mL distilled water (1:15 solid-to-liquid ratio, *w*/*v*) at room temperature (RT) for 1 h, followed by 1 h of draining on a mesh screen. The final weight was recorded in order to quantify the rehydration capacity (RHC) and the rehydration yield (RHY), which can be calculated using Equations (4) and (5) as follows:

(4) $$\mathrm{RHC}\;\left(\frac{g\;H_{2}O}{g\;\mathrm{sample}}\right)=\frac{\left(\mathrm{Weight}\;\mathrm{after}\;\mathrm{rehydration}\;(g)-\mathrm{Weight}\;\mathrm{before}\;\mathrm{rehydration}\;(g)\right)}{\mathrm{Weight}\;\mathrm{before}\;\mathrm{rehydration}\;(g)}\;$$

(5) $$\mathrm{RHY}\left(\%\right)=\left[\frac{\mathrm{Weight}\;\mathrm{after}\;\mathrm{rehydration}\;(g)-\mathrm{Weight}\;\mathrm{before}\;\mathrm{rehydration}\;(g)}{\mathrm{Weight}\;\mathrm{before}\;\mathrm{rehydration}\;(g)}\right]\;\times 100\;$$

#### 2.7.5. Cooking Loss

Minor modifications were made to the previous method described by Hong et al. [19] to conduct a cooking loss test. Ten grams each of TVPs were selected randomly soaked, cooked, and weighed using an analytical balance (Sartorius, ED224S, Goettingen, Germany) directly. After cooking, the samples were left at room temperature until the ambient temperature. The cooking loss was performed in six replications and was calculated as Equation (6) follows:

(6) $$\mathrm{Cooking}\;\mathrm{loss}\;\left(\%\right)=\frac{\mathrm{Initial}\;\mathrm{weight}\;(g)\;-\;\mathrm{Cooked}\;\mathrm{weight}\;(g)}{\mathrm{Initial}\;\mathrm{weight}\;(g)}\times 100$$

#### 2.7.6. Bulk Density

Minor modifications were made to the previous method described by Hong et al. [19] to conduct a bulk density test. TVPs were poured into a 1 L graduated cylinder and tapped twice to eliminate the spaces between the crumbles. The mass and volume were recorded, and the bulk density was determined by dividing the mass by the volume (g/L). There were two measurements taken for each sample.

### 2.8. Textural Properties Measurement

Texture profile analysis (TPA) of soaked or uncooked and cooked TVPs was measured in the test setting parameters of TA.XT Plus Texture Analyzer (Stable Micro Systems, Surrey, UK) according to Hong et al. [19]. For sample preparation, TVP analogs and TVP-Com was soaked in distilled water in the ratio 1:1 (*w*/*v*) for 1 h (soaked/uncooked) and cooked by pan searing without oil for 15 min. (cooked) before measurement. Uncooked and cooked samples (12 replications) were sheared using two cycles of compression using a cylinder probe (SMSP/36R, cylinder diameter = 36 mm). The testing conditions were as follows: pre-test speed = 1 mm/s; test speed = 5 mm/s; post-test speed = 5 mm/s; strain = 50%; trigger force = 10 g; the time interval between the two compressions: 5 s. The results were calculated by the EXPONENT CONNECT® software (Stable Micro Sys-tems, Surrey, UK) as hardness (g), chewiness (g·s), springiness (%), adhesiveness, cohesiveness, gumminess, and resilience (%). Firmness (g) and toughness (g·s) were also determined with the same setting condition with the strain mode. The analyses were performed in twelve replicates for each sample and expressed as the means.

### 2.9. Thai Northern-Style Sausage (Sai-aua) Preparation

The TVP analogs, TVP-KO, TVP-PH, and TVP-Com were used as meat substitutes for producing Sai-aua. TVPs were soaked in water (1:15 solid-to-liquid ratio, *w*/*v*) for 1 h at room temperature (RT), followed by draining for 1 h on a strainer. Each soaked TVPs (80 wt%) were mixed with Sai-aua chili paste (15 wt%) and seasoning (5 wt%) in the mixing bowl. The mixture was homogenized manually by hand and then filled into cellulose casings (diameter 3.5 cm and 19 cm in length). The Sai-aua from TVPs were cooked by steaming for 5 min followed by grilling at 140 °C for 12 min. using an electric oven (OTTO, CO-707, Supor Co., Ltd., Hangzhou, China).

#### Sensory Analysis of Plant-Based Sai-aua

According to COE no. 126/2022, ethical review and approval were waived for this study. The Mae Fah Luang University Ethics Committee on Human Research (MFU EC) reviewed Protocol No. EC 21191-14, in accordance with international guidelines such as the Declaration of Helsinki, the Belmont Report, CIOMS Guidelines, and the International Conference on Harmonization of Technical Requirements for the Registration of Pharmaceuticals for Human Use—Good Clinical Practice (ICH-GCP) and decided to exempt the aforementioned research protocol on 19 September 2022. The sensory evaluation focused on appearance, color, odor, flavor, texture, firmness, juiciness, and overall acceptability. The scoring of each sample was performed according to a 9-point hedonic scale to assess consumers’ preferences for each attribute [20]. On the 9-point hedonic scale, ‘9’ corresponded to ‘strongly like’ and ‘1’ corresponded to ‘strongly dislike’. A mean liking on a nine-point scale is usually indicative of highly acceptable sensory quality; hence, a product achieving this score could be used confidently as a suitable illustration of ‘target’ quality [20]. To mimic the consumers’ evaluation more objectively and actually, 30 untrained panelists (consisting of 15 females and 15 males, aged 18–65 years) were recruited from Mae Fah Luang University, Chiang Rai province, Thailand, randomly. The Sai-aua analogs were warmed at 140 °C for 5 min using an electric oven before assessment. Samples were kept warm (37 °C) in a heater for between 5 and 10 min until evaluation. The plant-based Sai-aua samples and commercial plant-based Sai-aua were cut into slices with a thickness of 1.5 cm and then distributed to panelists. The commercial plant-based Sai-aua was used as a benchmark. Samples were given to panelists one at a time in an order that was established to avoid the effect of sample order presentation. Panelists were asked to drink some drinking water to rinse their palate among samples. A sensory session of Sai-aua analogs was performed at 22 ± 1 °C in isolated rooms (individual cabins) under controlled environmental conditions with white light (300 lx) and relative humidity of 54 ± 1%. Moreover, to avoid the impact of shocks, all panelists were informed in advance that the samples were a novel product designed to replace conventional animal meat Sai-aua.

For the just-about-right (JAR) scale, the appropriateness of the intensities of seven sensory attributes including mushroom flavor, beany, chili paste taste, firmness, juiciness and Sai-aua-like texture, was evaluated by ratings provided on a 5-JAR scale, where 1 and 2 corresponded to “too weak” (TW), 3 to “just about-right” (JAR) and 4 and 5 to “too strong” (TS) evaluations according to Mörlein [21].

### 2.10. Statistical Analysis

All statistical data were analyzed with one-way ANOVA and Duncan’s multiple range test using IBM SPSS 16.0 (Version 16.0, IBM Corporation, Armonk, NY, USA). A *p*-value < 0.05 was considered statistically significant. Values are presented as mean ± standard deviation based on at least three independent analyses except for the amino acid composition. Twelve and ten replicates were executed for texture profile and color analysis, respectively.

## 3. Results and Discussion

### 3.1. Nutritional Compositions of Some Edible Mushrooms

Edible mushrooms were formerly called the ‘meat of poverty’ because of mass-scale production, availability, and affordable price. Edible mushrooms are rich in nutrients, including proteins, essential minerals and vitamins, complex polysaccharides, essential unsaturated fatty acids, and secondary metabolites [7,8]. In this study, the selected mushrooms were recruited by availability, affordable price, nutritional value, and health benefits.

The results of the nutritional value obtained for the studied edible mushrooms are shown in Table 2. From the screening, carbohydrate (57–62 wt%) were and high while the contents of ash (2.67–7.43 wt%) and fat (0.47–2.0 wt%) were low in all mushrooms. Mushrooms contain a high proportion of moisture and depend on the mushroom species and other factors such as harvesting, growth, and storage conditions [22]. Each mushroom presented similar amounts of proteins (23–29 g/100 g), fat contents, and carbohydrates values except those of white jelly and black fungus mushroom. Since, white jelly and black fungus mushrooms were retrieved in dried form from the shelf and assumed to have a long shelf life before analysis. Dimopoulou et al. [23] document that 16 mushroom species of protein ranged from 13.8 to 38.5 g/100 g, carbohydrate ranged 32 to 61.4 g/100 g, fat ranged from 0.4 to 5.9 g/100 g and ash ranged 1.3 to 14.4 g/100 g.

Mushrooms are reported to be a suitable source of protein. From the study, Pheonix mushroom showed the highest protein content (29.29 wt%) followed by King Oyster, black Shimeji, Enoki, white Shimeji and white jelly and black fungus (23.25, 23.06, 22.02, 20.71, 9.09, and 9.38, wt%, respectively). Tepsongkroh et al. [24] reported the protein contents *P. ostreatus* (22.01 wt%) and *P. pulmonarius* (21.16 wt%). The current results agreed with Reis et al. [22] and Sardar et al. [25]. The crude protein of *Pleurotus eryngii* in the range of (18.93–25.36 wt%) was reported according to Sardar et al. [25]. The protein content of mushrooms is typically high but varies widely based on factors such as the genetic profile of the species, the compost or growing medium, and the harvesting stage [24,25].

Carbohydrates, calculated excluding protein, ash, and fat levels, were the most abundant macronutrients, and the highest level was found in white jelly and black fungus that provided the highest carbohydrates level (72.24 and 76.48 g/100 g, d.b., respectively). Higher carbohydrate content is likely the result of a greater proportion of non-fiber carbohydrates, such as sugars, as well as fiber, structural polysaccharides, beta-glucans, chitin, hemicelluloses, and pectic substances [26]. Tepsongkroh et al. [24] reported the highest amount of carbohydrate was obtained from *P. pulmonarius* and *P. ostreatus*, at 56.78 and 56.30 wt%, respectively. Due to the presence of non-starch polysaccharides, mushrooms are a potential source of dietary fibers. In all cases, the stem portion of the mushroom contained more insoluble dietary fibers than the pilei [27]. In this study, KO and PH contained total dietary fiber accounting for 32.63 and 45.73 wt% (Table 2). The total dietary fiber in mushrooms consists primarily of the non-digestible carbohydrate chitin [27]. Cuptapun et al. [28] and Tepsongkroh et al. [24] reported that the fiber contents of *P. pulmonarius* and *P. ostreatus* as 12.29–16.68% and 7.80–14.01%, respectively.

Enoki, white Shimeji, and black Shimeji revealed the highest ash (7.36–7.43 g/100 g) and fat (1.37–2.00 g/100 g) contents; white jelly and black fungus provided the lowest fat levels (0.46–0.47 g/100 g). In general, mushrooms are low-calorie foods since they provide low amounts of fat (Table 2). Tepsongkroh et al. [24] reported that the ash content of various mushrooms ranged from 5.27% to 9.48%, indicating that the mushrooms contained nutrient-rich minerals such as potassium and phosphorus. According to Reis et al. [22], the main components of mushroom ash are potassium, phosphorus, or magnesium, in addition to calcium, copper, iron, and zinc.

Mushroom are low in fat content ranging from 0.47 to 2.00 wt% in selected species (Table 2). Elavarasan [27] reported some essential fatty acids in fat of mushroom. Nonetheless, mushrooms are not considered to be a significant source of essential fatty acids for fulfilling the human body requirements. Due to a low total lipid content and a small proportion of desirable n-3 fatty acids, the nutritional value of mushroom lipids is limited [24]. In the case of *Pleurotus* species, oleic acid and linoleic acid are reported as the predominant monounsaturated and polyunsaturated fatty acids, respectively. Literature demonstrates discrepancies in the concentrations and types of fatty acids present in specific edible species, depending on the species and their geographic distribution, a factor that influences their nutritional value [26,29].

**Figure 1:**
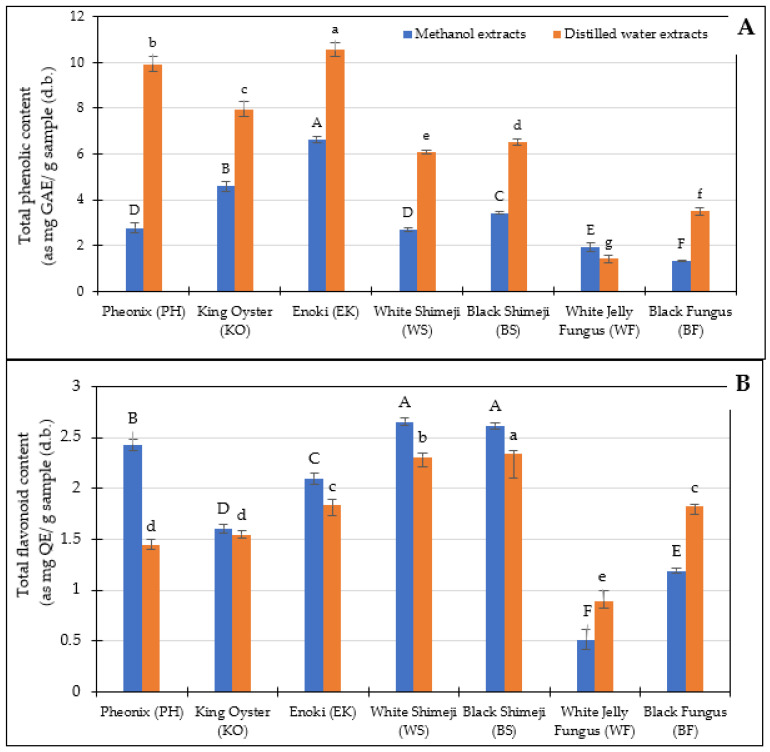
Total phenolic (**A**) and total flavonoid contents (**B**) of the seven different mushrooms. Bars represent the standard deviation. Different lowercase and uppercase letters indicate significant differences (*p* < 0.05) among mushroom extracts extracted from methanol and distilled water, respectively.

**Figure 2:**
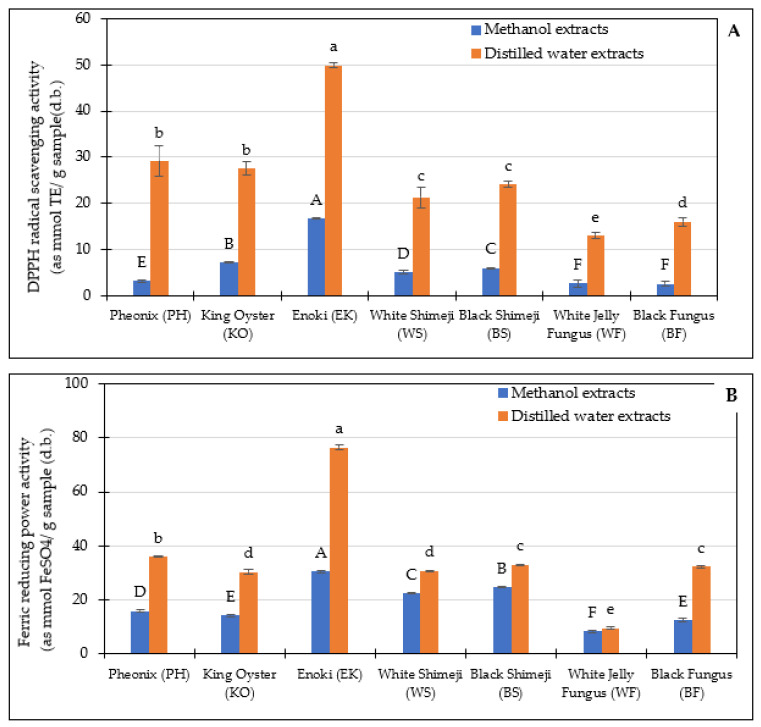
Antioxidant activities under DPPH radical scavenging activity (DPPH, (**A**)) and ferric reducing power activity (FRAP, (**B**)) of the seven different mushrooms. Bars represent the standard deviation. Different lowercase and uppercase letters indicate significant differences (*p* < 0.05) among mushroom extracts extracted from methanol and distilled water, respectively.

Only two types of mushrooms, both *Pleurotus* species (King Oyster (KO) and Pheonix (PH) mushrooms), were selected to determine amino acid compositions regarding higher protein content, bioactive compounds, and bio-properties (Figure 1 and Figure 2), as well as availability, the production scale, and cost. According to reports, mushrooms are a suitable source of protein, and some researchers have even argued that their amino acid profiles are comparable to those of animal proteins, as presented in Table 3 [22]. KO showed inferior amino acid content than that of PH mushrooms. The major essential amino acids in KO and PH are listed as leucine (1617.33 and 1897.47 mg/100 g, d.b.), lysine (1329.64 and 1575.17 mg/100 g, d.b.), threonine (1081.28 and 1367.32 mg/100 g, d.b.), valine (1398.27 and 1685.61 mg/100 g, d.b.), whereas glutamic acid (2855.36 and 6302.33 mg/100 g, d.b.), aspartic acid (1747.43 and 2331.38 mg/100 g, d.b.), alanine (1700.02 and 2045.82 mg/100 g, d.b.), glycine (1084.61 and 1329.72 mg/100 g, d.b.), and serine (1126.27 and 1508.64 mg/100 g, d.b.) are the most abundant non-essential amino acids, respectively. Methionine was the very limiting amino acid (217.77 and 287.18 mg/100 g, d.b.). It is known that the growth medium and condition can influence the chemical composition and, consequently, the cultivated mushrooms’ nutritional value [25]. Mushroom quality is also influenced by other parameters, such as the stage of development and pre- and post-harvest conditions [22]. In addition to intraspecific genetic variation, geographical origin, and producer affected the chemical composition and nutritional value of samples [22].

### 3.2. Bioactive Compounds and Bioactivities of Some Edible Mushrooms

#### 3.2.1. Total Phenolic and Flavonoid Content

In this study, two extracting solvents, distilled water and absolute methanol, were used to extract TPC and TFC from mushrooms. TPC and TFC of mushroom extracts were shown in Figure 1A,B, respectively. In the case of selected mushrooms, the TPC values ranged from 1.50 to 10.56 mg gallic acid equivalents (GE)/g sample (d.b.). All analyzed mushrooms extracted by distilled water showed higher TPC on average, 1- to 3-fold higher than that of methanol. The highest content of TPC in water extract of 10.56 mg GE/g sample, d.b. was observed in Enoki mushroom followed by Pheonix and King Oyster mushroom as 9.93 and 7.96 mg GE/g sample, d.b., respectively. These results could be explained by phenolic compounds are often extracted in higher amounts in more polar solvents. Thus, a higher TPC was observed. The highest TPC derived from hot water extracts in *Pleurotus* spp. between 4 and 80 µg GE/g sample was observed according to Tepsongkroh et al. [24]. Similar findings were reported by González-Palma et al. [30], who discovered that the TPC in several edible Indian mushrooms extracted with hot water was greater than that extracted with methanol.

The opposite trends were observed in TFC values in mushroom extracts by distilled water and methanol. The content of flavonoids found in all samples was low. The values were in the range of 0.50 to 2.65 mg quercetin equivalents (QE)/g sample, d.b. The highest content of TFC was observed in the methanolic extracts of black Shimeji as 2.65 mg QE/g sample (d.b.), followed by that of white Shimeji and PH mushroom as 2.61 and 2.43 mg QE/g sample (d.b.). Methanolic extracts presented superior TFC than that of water extracts. It should be because of the different polarity of solvent and flavonoid compounds’ structure, which solute more in methanol. Sudha et al. [31] reported that, specifically, the methanol extracts of various species of *Pleurotus* showed higher TFC with values of 6.38 to 7.79 mg catechin equivalents/g extract. The results indicated that the TPC and TFC vary depending on the mushroom species and extractant medium (*p* < 0.05).

#### 3.2.2. Antioxidants Properties

Antioxidant and ferric-reducing properties are depicted in Figure 2A. Consequently, antioxidant activity was also higher for water extracts, which concordance with the TPC content. Water extracts imparted polarity to compounds with a high number of hydroxyl groups, which acted as hydrogen donors [26]. Mushroom extracts exhibited different antioxidant capacities depending on species and extraction medium (13.03–29.07 and 3.52–16.64 as mmol Trolox equivalents (TE)/g sample (d.b.)) for DPPH activities of water and methanol extracts, respectively, and 30.84–76.54 and 8.36–30.54 as mmol FeSO_4_ equivalents (FE)/g sample (d.b.) for FRAP of water and methanol extracts, respectively. The top three ranking of DPPH values were found in water extract of Enoki, phoenix, and King Oyster mushroom as 49.91, 29.27, and 27.49 as mmol TE/g sample (d.b.) and FRAP were 76.54, 35.59, and 30.43 mmol FE/g sample (d.b.)., respectively. The higher antioxidant activity value on selected species of uncooked oyster mushrooms, *Pleurotus* spp. of 48.87 to 92.51 μmol TE/100 g by Tan et al. [32]. However, Ng and Tan [33] reported lower FRAP in selected raw edible mushrooms such as Pheonix, King Oyster, and Enoki as 11, 11, and 51 µmol FE/100 g FW, respectively, than those in the current study.

The different antioxidant properties can be explained by the different phenolic compounds’ structure represented in mushroom extract, which can be dissolved differently due to the different polarities of the solvents used [31]. The results indicated that water is a better extraction solvent for phenolic compounds of mushroom in the present study and, as a consequence, water extracts of these mushrooms have better antioxidant properties.

### 3.3. Physicochemical Analysis of Difference Mushroom-Based TVPs

#### 3.3.1. Color Analysis

Color is considered the most important quality characteristic in meat and is tightly associated with the quality of food and acceptance by consumers [10]. Table 4 shows the difference between TVP analogs and TVP-Com. L* represents the distinction between the color and the whiteboard. The greater the value, the smaller the color difference between the sample and the whiteboard, indicating that the surface was brighter. From the study, TVP-Com showed a significant difference in color parameters with TVP-KO and TVP-PH (L* = 54.58 ± 0.41, a* = 4.68 ± 0.23, and b* = 22.61 ± 0.65), while those of TVP analogs were similar between TVP-KO and TVP-PH. The lower lightness of TVP analogs value L* = 31.58 ± 0.50 for TVP-KO and 34.95 ± 0.67 TVP-PH because the color of the mushroom itself that is coloration to be the reddish tone by beet juice powder and together with the cooking processing that used high temperature in drying induce brown color by Maillard reaction. The slightly lower lightness of TVP-KO than TVP-PH due to the gray color of PH. a* and b* was lower in TVP analogs than those of TVP-Com due to the color of the ingredients. TVP-Com consisted of soy flour (either soy flour (50 wt% protein) or soy protein concentrate (70 wt% protein)) mixed with water, sodium chloride, and other ingredients, which present white or faint yellow powder [12].

#### 3.3.2. Visual Surface, Macrostructure, and Microstructure

**Figure 3:**
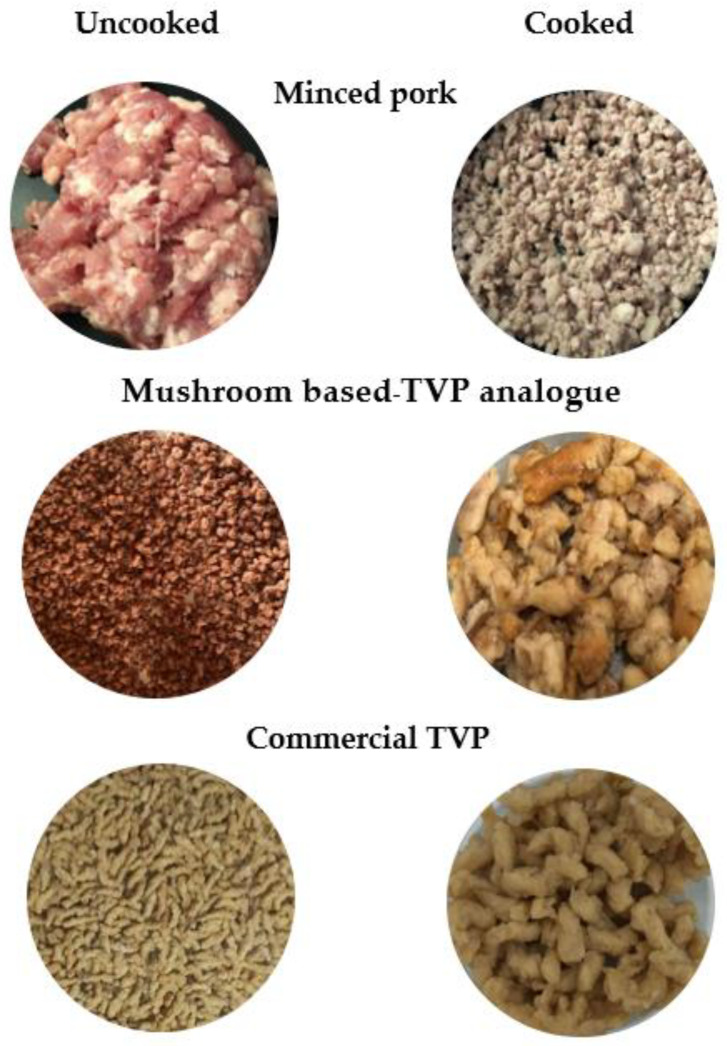
Visual appearance of minced pork, mushroom-based TVP analog, and commercial TVP before (**left**, uncooked) and after (**right**, cooked) cooking by searing in a non-stick frying pan without oil for 15 min.

**Figure 4:**
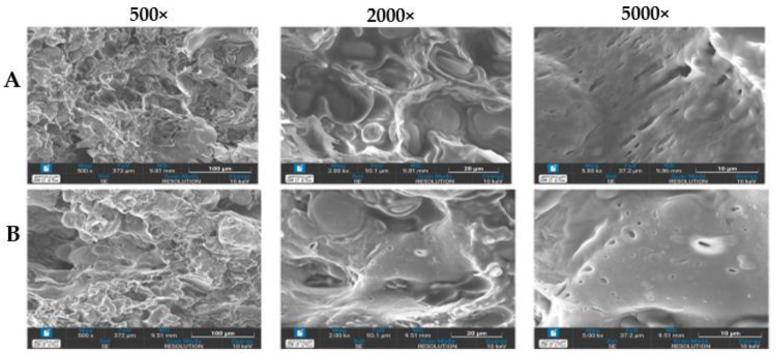
Microstructure of meat-based TVP analogs made from King Oyster mushroom (TVP-KO, (**A**)) and Pheonix mushrooms (TVP-PH, (**B**)) at a magnification of 500×, 2000×, and 5000×.

The TVP-KO and TVP-PH presented similar granules or tiny chunks of about 3 mm, whereas TVP-Com presented long granules of about 4–5 mm in length and 2 mm in width. The colors of TVP analogs look reddish and brown mixed, but when soaking and cooking, the color disappears and is disclosed to brown and similar to the color of cooked TVP-Com (Figure 3), which correlates to the color attributes in Table 4. As shown in Figure 3, the development of TVP analogs from mushrooms had an apparent effect on the macrostructure of TVPs, as can be observed in Figure 4A,B. The visual surface and macrostructure of TVP analogs, TVP-KO and TVP-PH, as illustrated in Figure 4, the surface of TVP-KO and TVP-PH became similar. However, at the highest magnification of 5000×, the fibrous structure with diagonal air cell was observed in TVP-KO. TVP-PH showed more air cell bubbles and less fibrous structure. Notably, the images of TVPs were captured in their naturally exhibited directions (longitudinal or horizontal cross-sections of extrusion) because the current samples from the experiment were difficult to cut intentionally due to their shape and size constraints. This may explain why TVP-KO had more elongated cells and TVP-PH had smaller pores. The limitation of texturizing plant protein by using a single-screw extruder was recognized as no anisotropic structure could be observed, similar to that produced by the standard single-screw or a twin-screw extruder [34,35].

#### 3.3.3. Water and Oil Holding Capacity (WHC and OHC)

The water-holding capacity (WHC) of a material represents its hydration properties and indicates the amount of water it can absorb at the macromolecular level [36]. WHC is known to play a significant role in the development of food texture, and higher levels of WHC result in less cooking loss, indicating WHC as a quality index for TVPs. As fresh raw meat is considered a high-moisture food, meat analogs must contain a higher WHC to mimic the texture, succulence (juiciness), and chewiness of authentic meat [19]. A protein’s amphiphilicity gives it the ability to interact with both water and oil. In addition, WHC and OHC depend on the presence of polar and non-polar amino acid residues, as well as the micro- and macrostructures of the protein [19]. Future research must clarify the amino acid profiles of TVP analogs in order to provide additional evidence.

Table 4 compares and summarizes particular functional properties of TVP analogs and TVP-Com. TVP-KO and TVP-PH demonstrated lower WHC (2.19, 2.09, and 3.45 g H_2_O/g sample) and OHC (1.23, 1.08, and 1.45 g oil/g sample) than TVP-Com. Low-moisture extrusion processing contributed to TVP-Com’s capacity to retain both water and oil. TVP expands rapidly upon emergence from the die and loses a great deal of water through evaporation, resulting in a sponge-like structure before being dried to safe moisture levels of 7–10% for storage [11].

TVP-Com had the highest OHC with 1.42 g oil per gram, followed by TVP-KO and TVP-PH with 1.23 and 1.08 g H_2_O per gram, respectively. WHC and OHC may correlate with the degree of denaturation since extrusion cooking causes the unfolding of proteins and the exposure of more hydrophobic sites [35]. TVP-Com is derived from extrusion cooking, a high-temperature, short-time (HTST) process. High temperature, high pressure, and mechanical shear cause protein denaturation, partial unfolding, and subsequent aggregation in proteinaceous food materials. The structural modifications induced by heat treatment result in the formation of a more flexible protein conformation, which may be considered the responsible factor for the improvement of surface activity that affects WHC and OHC [37], thereby introducing more accessible hydrophobic sites that contribute to higher OHC values in TVP-Com. The heating of proteins during extrusion cooking may change the distribution pattern of hydrophilic and hydrophobic sites on the protein surface and expose the functional groups, which consequently affect their surface activity and facilitates the absorption and formation of a physical layer at air-water and oil-water interfaces [38]. In addition, polar carbohydrates may have a detrimental effect on the extent of oil interactions. According to Osen et al. [35] and Samard et al. [39], the relationship between carbohydrate content and OHC is inverse.

The absorption of water, oil content, and meat-like texture of TVP also depends on the raw material used to produce it [11]. Utilizing the appropriate formulation and processing techniques can aid in achieving these properties. To produce TVP, soy flour with a protein content of 50% and a protein dispersibility index (PDI) of 60 to 70 is recommended. In the production of soy concentrates, a material with a lower protein solubility can be utilized. Other ingredients may be added to a recipe for a variety of reasons, including economics, nutritional balance, functionality, color of the final product, or allergen reduction. Vital wheat gluten, which is highly soluble in water and contains protein of at least 80 wt%, can be combined with soy flour or soy concentrate to produce TVP [11].

#### 3.3.4. Water Activity (a_w_)

a_w_ below 0.7 inhibits microbial growth, and the material can be considered storage stable [40]. TVP-Com exhibited the lowest a_w_ of 0.65, followed by TVP-PH and TVP-KO with 0.84 and 0.90, respectively. This proved that TVP could be a stable shelf life because of their low moisture and water activity. After extrusion processing, TVP is conveyed to a dryer where long retention times are required to remove water, thus it from the dense structure, thereby leading the low moisture and a_w_ in TVP. TVP is not only for Niche markets such as vegetarian, vegan, and flexitarian. It fulfills food scarcity, esp. in war zone areas and under-developing countries, due to its being shelf stable, lightweight, and nutritional.

#### 3.3.5. Rehydrating Capacity and Yield

Water is essential in meat products for imparting the proper texture and juiciness to ensure customer acceptance. Rehydrating capacity (RHC), which refers to the amount of water that intact TVP could hold upon rehydration, is a crucial factor influencing the meat-like chewiness of plant-based meat analogs [41]. As shown in Table 4, the RHC values of TVP-KO, TVP-PH, and TVP-Com were significantly different in the current study, ranging from 1.5 to 4.2 g/g. The rehydrating yields of TVP-KO, TVP-PH, and TVP-Com were 102.07 ± 0.53, 108.05 ± 0.65, and 187.65 ± 0.02, respectively, indicating that the weight of rehydrated TVP was greater than that of dried TVP prior to soaking. The retain ability was very high in TVP-Com, proving the better heat and processing by the standard manufacturer. Differences in RHC are dependent on protein types, protein-water molecule interactions, and water-water molecule interactions but are more closely related to product structure, particularly porosity and air cell size (Figure 4) [19].

To transform TVP into a ready-to-eat meat alternative, it must absorb at least three times its weight in water, which takes 15 to 20 min under boiling conditions or as long as several hours in cold water, then can be used for further processing into ready-to-eat meals and as meat extenders [11]. The texture becomes chewy and juicy upon rehydration to mimic the texture of meat [42]. TVP made with soy flour can typically absorb 2.5 to 3 times their weight in water, whereas those made with soy concentrates can absorb 4.5 to 5 times their weight in water. Rehydration rates are proportional to product size and surface area [11]. The rehydration yields of TVP samples are also represented in Table 4. This highest rehydration yield was observed from TVP-Com (187.65%), whereas that of TVP-KO and TVP-PH was 102–108%. From the results, it could be mentioned that TVP-Com yields a better value because of its spongy structure, which permits faster water absorption and a higher amount of water [11]. In addition to the number of pores, the size of the air space is crucial for water retention [19]. Contrary to some prior research that linked a greater RHC to a lower bulk density, goods with a low bulk density may have a higher porosity (TVP-Com), which permits faster water absorption and hence a higher WHC.

#### 3.3.6. Cooking Loss

Cooking loss is of interest because it is expected to explain part of the variation in juiciness but also because it influences the appearance of the meat [43]. The cooking loss of TVP-KO 33.88 ± 0.02% was lower than that of TVP-PH 35.53 ± 0.05%. TVP-Com showed the highest cooking loss, 63.58 ± 0.02%. The study showed a small loss of moisture and retained juiciness in TVP analogs. The parameters influence the cooking loss, and part of the effect on juiciness might depend on the raw meat quality center temperature cooking procedure (heating time/heating temperature and heating method). In real meat, it has been shown that juiciness and cooking loss are negatively correlated, implying that a high cooking loss results in low juiciness [43]. TVP-Com showed the highest cooking loss, which is associated with their high porous structure. TVP has a porous structure owing to their expansion and has a sponge-like structure that rapidly absorbs water; however, the more pores, the more total volume of water can penetrate and be adsorbed [11].

#### 3.3.7. Bulk Density

The bulk density of TVP products interprets the overall expansion and changes in the protein network [44]. The studied TVP samples displayed a wide range in bulk density, as shown in Table 4, with TVP-PH being the highest (0.50 g/L) and TVP-Com being the lowest (0.28 g/L). TVP-KOs derived exhibited (0.44 g/L). Conventionally, higher protein content has been shown to undergo a higher degree of protein cross-linking and forming strong structures, which prevents expansion, thus increasing bulk density [45]. However, in this case, the varied inherent qualities of the raw material may contribute more to bulk density. As noted previously, in comparison to other proteins, soy protein often possesses superior texturizing properties and forms stronger structures, resulting in a greater bulk density, esp. TVP-Com. In addition, additional extrusion processing variables, including feed moisture, extruder barrel temperature, and screw speed, may also contribute to the broad range of bulk density [1,13,19].

### 3.4. Texture Analysis of Difference Mushroom-Based TVPs

Texture is unquestionably the most important factor in determining the quality of textured plant proteins since the primary objective of meat substitutes is to emulate the desired texture of real meat [19]. The research demonstrated that TVPs consisting of a mixture of protein powder, mushroom powder, and water made from a single-screw cold extrusion process could induce the formation of a spongy and chewy texture (Figure 4). Textural profile analysis (TPA) simulates chewing using a double compression test and generates sensory-relevant characteristics from the force–time curves that follow [46].

**Figure 5:**
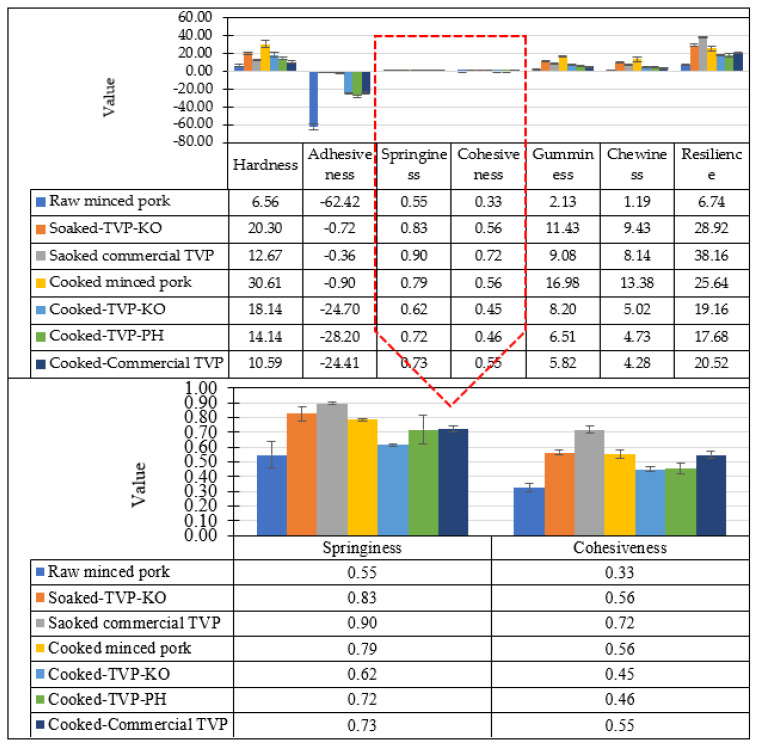
Textural properties of raw and cooked TVP analogs made from King Oyster mushroom (TVP-KO) and Pheonix mushrooms (TVP-PH), and commercial TVP (TVP-Com) reported as hardness (N), chewiness (N.s), adhesiveness (%), springiness, cohesiveness, gumminess, and resilience (%) from 12 replications. Bars represent the standard deviation.

Textural properties of uncooked and cooked TVP analog, TVP-Com, and minced pork are shown in Figure 5. The uncooked version provides a lower value than the cooked version except for adhesiveness. In raw minced meat, the hardness and chewiness increase upon cooking, while all TVP was lower in those values due to the immersion in water, making TVP soft. The best resilience was observed in TVP-Com since it derived from the high-temperature extrusion process, which contributes to the porousness to hold water and fluffy structure [19,47].

Protein-rich raw materials are more easily texturized with less energy input using an extruder. In addition, commodities with a higher protein content tend to have harder and firmer textures [47]. Fiber from mushrooms 32.63 and 45.73 g/100 g, in PH and KO, respectively, could have a negative effect on the texturization of protein. As well, without the high heat during extrusion processing, the desired structure could not be formed well. The combination of heating and shearing during extrusion cooking of TVP-Com leads to a porous sponge-like structure owing to their expansion [47]. Additionally, according to Riaz [11], a high fiber content will hinder the texturization process by diluting the protein concentration and generating discontinuities in the texturized matrix. The cross-linking of protein macromolecules is somewhat inhibited by fiber, which might influence the structure and texture of the texturized product. This could be the reason why TVP analogs and TVP-Com are harder and have a softer texture.

### 3.5. Quality Parameters of Thai Northern-Style Sausage from Mushroom-Based TVP

**Figure 6:**
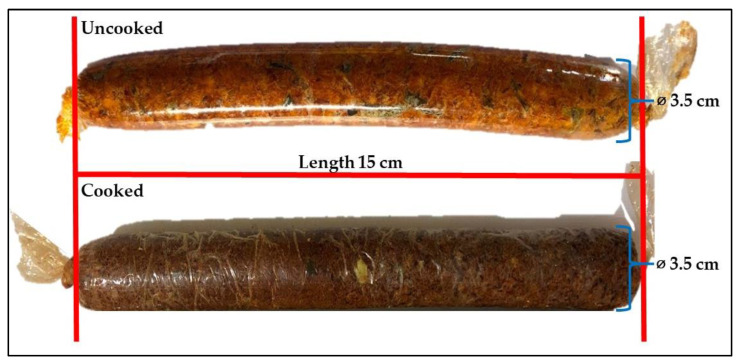
Visual appearance of uncooked and cooked Thai Northern-style sausage (Sai-aua) made from mushroom-based texturized vegetable protein (TVP) in cellulose casing (diameter 3.5 cm and 15 cm in length).

Though recent consumer trends have accepted the concepts of sustainability and wellness, the sensory attributes, especially taste, and texture, of food products are one of the most important factors that consumers consider when deciding whether to purchase or to re-purchase. Thus, the development of Thai Northern traditional dishes from TVP analogs was invented to meet consumers’ expectations and to mimic the quality characteristics of animal-based Sai-aua. The visual appearance of the Sai-aua analog is depicted in Figure 6.

**Figure 7:**
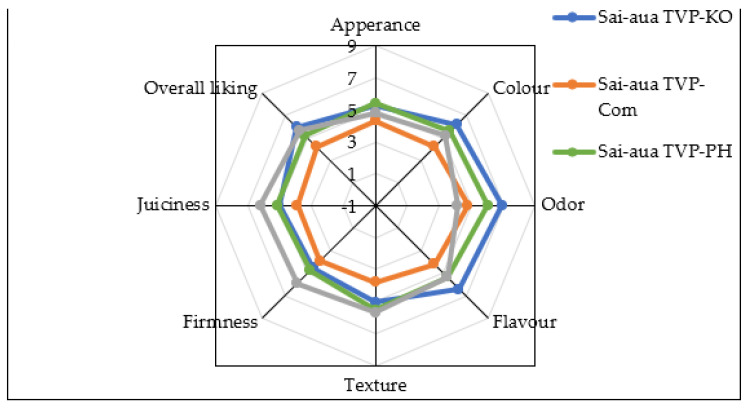
Sensory profile of Thai Northern-style sausage (Sai-aua) made from King Oyster mushroom-based TVP (Sai-aua TVP-KO, blue line) and Pheonix mushroom-based TVP (Sai-aua TVP-PH, green line), and commercial TVP (Sai-aua TVP-Com, orange line) evaluated compared to commercial plant-based Sai-aua (gray line) by 30 untrained panelists.

For sensory attributes, appearance, color, odor, flavor, texture, firmness, juiciness, and overall acceptability were presented in Figure 7. Sai-aua TVP-KO gained the best overall acceptability, followed by Sai-aua TVP-PH and Sai-aua TVP-Com with the score of 5.95 ± 0.93, 5.20 ± 0.62, and 4.20 ± 0.81, respectively whereas 5.65 ± 0.26 for commercial plant-based Sai-aua (Figure 7). Form the high score mainly contributed the study, the highest overall acceptability observed in Sai-aua from TVP analogs for attributes such as appearance, color, odor, and flavor. The results confirm the potential of using TVPS for the development of plant-based meat products with satisfied sensory qualities and high acceptance from consumers. TVP-KO proved the highest and most comparable to commercial plant-based Sai-aua, whereas TVP-Com Sai-aua presented the lowest score of acceptance. However, the texture, juiciness, and firmness of Sai-aua from TVP analogs need to be improved in future work. The result from this stage can be applied in future work to develop Sai-aua analogs to meet consumers’ expectations.

**Figure 8:**
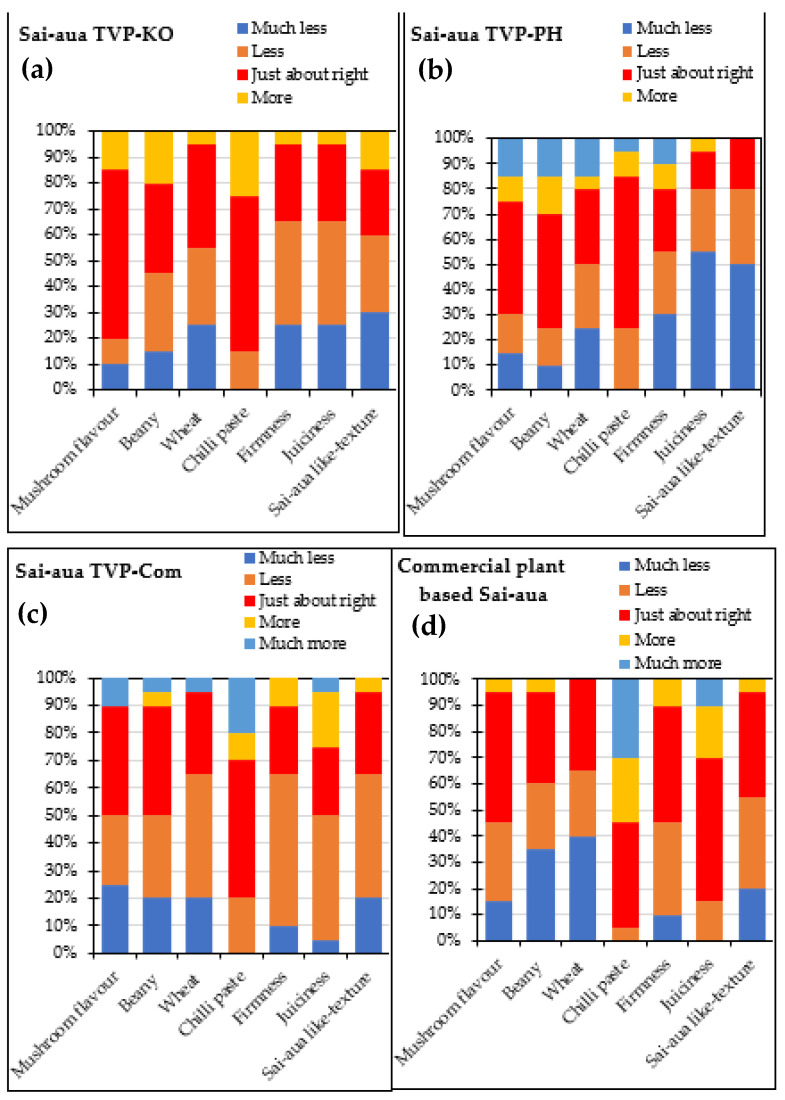
Just-about-right (JAR) scales of Thai Norther--style sausage (Sai-aua) analogs made from King Oyster mushroom-based TVP (Sai-aua TVP-KO, (**a**)) and Pheonix mushroom-based TVP (Sai-aua TVP-PH, (**b**)) and commercial TVP (Sai-aua TVP-Com, (**c**)) evaluated compared commercial plant-based (**d**) Sai-aua by 30 untrained panelists.

From the JAR scale, panelists were asked to evaluate the optimum in different aspects such as mushroom odor, beany odor, wheat odor, chili paste, juiciness, firmness, and Sai-aua-like texture of Sai-aua analog. In commercial plant-based Sai-aua, all the parameters were mainly scored in the “JAR” category, ranging from 35% (beany-wheat odor) to 45% (juiciness). The most remarkable results were that 40–55% of the consumers thought that the firmness, Sai-aua-like texture, and juiciness of the commercial plant-based Sai-aua were “JAR”. The Sai-aua analog samples showed lower percentages of JAR responses than the commercial plant-based Sai-aua for most of the attributes evaluated (Figure 8), and it was observed that the percentage of JAR responses dropped as in texture attributes. The developed Sai-aua analogs showed higher “JAR” in taste attributes (mushroom, beany, wheat odor, and chili paste) than those of commercial plant-based Sai-aua.

Consumers considered Sai-aua TVP-Com, and Sai-aua TVP-PH to have “much more” mushroom, beany, and wheat odor (5–10%) and (15%), respectively. The obtained results also showed that the TVP-Com might have its unique odor, and PH mushroom decreased the perception of Sai-aua TVP-PH odor. Rothman [48] documented that JAR provides guidance for product reformulation or a better understanding of attribute adequacy in relation to liking in terms of direction, with the assumption that the maximum hedonic score will occur at the JAR point. Thus, Sai-aua analogs from mushroom-based TVP will be reformulated and developed to acquire the optimum recipe and best consumer preference in future studies.

## 4. Conclusions

King Oyster and Phoenix mushrooms were selected to use as the main ingredients due to their higher nutritional values and bioactivity, as well as their availability and affordable price. With the right proportions of mushroom and other ingredients, the invented TVPs from conventional single-screw extrusion could maintain a structure, chewiness, and juiciness comparable to that of minced pork. Even though mushroom-based TVPs showed inferiority in certain properties such as WHC, OBC, RHC, and hardness compared to TVP-Com, they had an attractive reddish color, high nutritional value, and were preferred by consumers. This study has demonstrated the viability of developing value-added products to meet consumer demand, as well as establishing a cost-effective processing technology (conventional single-screw extrusion with natural additives) that could benefit both manufacturers and consumers by minimizing the price differential between meat substitutes and real meat. This study demonstrates the potential of mushrooms as an innovative alternative ingredient for the production of TVP, as well as their economic and ecological sustainability. The results may provide future scientific references for conventional processing procedures and the development of meat substitutes.

## Figures and Tables

**Table 1:** Ingredients of commercial plant-based meat and TVP analogs.

Ingredients (g)	Commercial Plant-Based Minced Meat	TVP Analogs
Water	55	30–35
Soy protein isolate	19	15–20
Mushroom: Split gill/King Oyster/Pheonix	17	30–35
Wheat flour or gluten	3	5–10
Canola oil	2	3–5
Coconut oil	1	0
Yeast extract	1	0.5–1
Beet juice powder	1	1–2
Chickpea flour	0	5–10
INS 407 Carrageenan	0.5	0
INS 461 Methyl Cellulose	0.5	0
Total	100	100

**Table 2:** Mean nutrient content of dried mushroom powders reported as g per 100 g or wt%.

Sample (g/100 g, wt%)	Moisture	Dry Matter	Protein	Ash	Lipid	Carbohydrate
Pheonix/Indian Oyster	4.61 ± 0.15 e	95.39 ± 0.15 a	29.29 ± 0.05 a	6.95 ± 0.07 c	1.39 ± 0.03 c	57.76 ± 0.22 e (Dietary fiber: 32.63)
King Oyster	8.16 ± 0.09 c	91.84 ± 0.19 c	23.25 ± 0.03 b	6.90 ± 0.09 c	0.73 ± 0.04 e	60.97 ± 0.07 c (45.73)
Enoki	7.34 ± 0.19 d	92.66 ± 0.19 b	22.02 ± 0.15 c	7.43 ± 0.08 a	1.37 ± 0.02 d	61.84 ± 0.20 c
White Shimeji	8.05 ± 0.06 c	91.95 ± 0.06 c	20.71 ± 0.17 d	7.40 ± 0.08 a	1.85 ± 0.17 b	61.99 ± 0.15 c
Black Shimeji	8.55 ± 0.07 b	91.45 ± 0.07 d	23.06 ± 0.11 b	7.36 ± 0.13 a	2.00 ± 0.06 a	59.05 ± 0.15 d
White Jelly Fungus	11.10 ± 0.08 a	88.90 ± 0.08 e	9.09 ± 0.04 f	7.11 ± 0.18 b	0.46 ± 0.05 f	72.24 ± 0.24 b
Black Fungus	11.05 ± 0.11 a	88.95 ± 0.11 e	9.38 ± 0.02 e	2.62 ± 0.02 d	0.47 ± 0.01 f	76.48 ± 0.10 a

Values represent mean observations of three replicates ± standard deviations. The mean values encoded by different lowercase letters in the same column are significantly different (*p* < 0.05).

**Table 3:** Amino acid contents of two potential mushrooms reported as mg/100 g sample (d.b.).

Amino Acid (mg/100 g, Dry Basis)	King Oyster	Pheonix
Essential amino acids		
Histidine	526.39	726.05
Isoleucine	858.90	970.69
Leucine	1617.33	1897.47
Lysine	1329.64	1575.17
Methionine	217.77	287.18
Phenylalanine	901.33	1275.33
Threonine	1081.28	1367.32
Tryptophan	261.18	418.61
Valine	1398.37	1685.61
Non-essential amino acids		
Glutamic acid	2855.36	6302.33
Aspartic acid	1747.43	2331.38
Alanine	1700.02	2045.82
Glycine	1084.61	1329.72
Proline	890.07	1011.94
Serine	1126.27	1508.64
Tyrosine	598.82	830.67
Arginine	907.38	1217.31
Cystine	ND	ND
Hydroxylysine	ND	ND
Hydroxyproline	ND	ND

ND = not detected.

**Table 4:** Physicochemical properties of difference mushroom-based and commercial TVPs.

Properties	TVP-KO	TVP-PH	TVP-Com
Proximate compositions (wt%, dry basis)			
Moisture	8.27 ± 0.07 a	8.09 ± 0.07 b	7.99 ± 0.06 b
Protein	45.59 ± 0.33 c	47.78 ± 0.24 a	47.29 ± 0.59 b
Ash	3.99 ± 0.33 c	4.54 ± 0.08 b	8.02 ± 0.04 a
Lipids	8.10 ± 0.19 a	7.89 ± 0.09 a	0.84 ± 0.06 b
Carbohydrates	33.86 ± 0.34 c	35.59 ± 0.16 b	39.73 ± 0.55 a
Water-holding capacity (g H_2_O/g sample)	2.19 ± 0.07 b	2.09 ± 0.08 b	3.45 ± 0.10 a
Oil-binding capacity (g Canola oil/g sample)	1.23 ± 0.09 b	1.08 ± 0.09 b	1.42 ± 0.05 a
Bulk density (g/L)	0.44 ± 0.04 b	0.50 ± 0.01 a	0.28 ± 0.01 c
Rehydration capacity (g/g)	0.96 ± 0.00 c	1.21 ± 0.04 b	2.08 ± 0.06 a
Rehydration yield (%)	102.07 ± 0.53 c	108.05 ± 0.66 b	187.65 ± 0.02 a
Cooking loss (%)	33.88 ± 0.02 c	35.53 ± 0.05 b	63.58 ± 0.02 a
pH	7.01 ± 0.01 a	7.09 ± 0.01 b	7.06 ± 0.01 c
a_W_	0.90 ± 0.01 a	0.84 ± 0.00 b	0.65 ± 0.00 c
Color parameters			
L*	31.58 ± 0.50 c	34.95 ± 0.67 b	54.58 ± 0.41 a
a*	9.30 ± 0.72 a	9.23 ± 0.37 a	4.68 ± 0.23 b
b*	16.92 ± 0.2 c	18.72 ± 0.40 b	22.61 ± 0.65 a

Means with different lowercase letters within the same row are significantly different (*p* < 0.05) among samples. The parameters L* indicated the lightness of the sample from blackness (L* = 0) to whiteness (L* = 100). The a* indicated red (a* is positive) and green (a* is negative) of the sample. The b* indicated yellow (b* is positive) and blue (b* is negative) of the sample.

## Data Availability

Data is unavailable due to privacy or ethical restrictions.
